# Isolation of extended‐spectrum β‐lactamase‐producing *Escherichia coli* from Japanese red fox (*Vulpes vulpes japonica*)

**DOI:** 10.1002/mbo3.1317

**Published:** 2022-09-19

**Authors:** Tetsuo Asai, Michiyo Sugiyama, Tsutomu Omatsu, Masato Yoshikawa, Toshifumi Minamoto

**Affiliations:** ^1^ Department of Applied Veterinary Sciences, United Graduate School of Veterinary Sciences Gifu University Gifu Japan; ^2^ Crisis Management Unit, Center for Infectious Diseases Epidemiology and Prevention Research Tokyo University of Agriculture and Technology Fuchu‐shi Tokyo Japan; ^3^ Division of Environment Conservation, Institute of Agriculture Tokyo University of Agriculture and Technology Fuchu‐shi Tokyo Japan; ^4^ Department of Human Environmental Science, Graduate School of Human Development and Environment Kobe University Kobe Hyogo Japan

**Keywords:** antimicrobial resistance, ESBL, fox, wildlife

## Abstract

Antimicrobial resistance is a global concern requiring a one‐health approach. Given wild animals can harbor antimicrobial‐resistant bacteria (ARB), we investigated their presence in 11 fecal samples from wild animals using deoxycholate hydrogen sulfide lactose agar with or without cefotaxime (CTX, 1 mg/L). Thus, we isolated CTX‐resistant *Escherichia coli* from two Japanese red fox fecal samples. One strain was O83:H42‐ST1485‐fimH58 CTX‐M‐55‐producing *E. coli* carrying the genes *aph(3″)‐Ib*, *aph(3′)‐Ia*, *aph(6)‐Id*, *mdf*(A), *sitABCD*, *sul*2, *tet*(A), and *tet*(B), whereas the other was O25:H4‐ST131‐fimH30 CTX‐M‐14**‐**producing *E. coli* carrying *mdf*(A) and *sitABCD* and showing fluoroquinolone resistance. Thus, the presence of extended‐spectrum β‐lactamase producers in wild foxes suggests a spillover of ARB from human activities to these wild animals.

## Graphical abstract

Wild animals are potential sentinels of antimicrobial‐resistant bacteria (ARB) in the environment, while infection in humans living in wildlife areas poses a potential risk of transmitting pathogens, including ARB. We report here that extended‐spectrum beta‐lactamase producers may have spilled over from humans and domestic animals. This suggests that handling dropped feces with care around areas of human activity is essential.

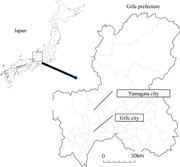

## 1 INTRODUCTION

Antimicrobial resistance (AMR) in bacteria represents a common global issue in humans, domestic animals, and the environment. The global action plan on AMR published by the World Health Organization (World Health Organization, 2015) states that multiple sectors, comprising humans, animals, and the environment, including wild animals, should be corroborated globally to control the emergence and prevalence of antimicrobial‐resistant bacteria (ARB). Multisectoral approaches have been implemented according to the national action plan on AMR in Japan, as described in the Nippon AMR One Health Report (The AMR One Health Surveillance Committee, 2021). Furthermore, in Japan, ARB in wild animals has been addressed as a component of the environment since 2020, and the prevalence of AMR among *Escherichia coli* found in several wild animals has been recently reported (Asai et al., 2020; Fukuda et al., 2021; Tamamura‐Andoh et al., 2021).

Wild animals are potential sentinels of ARB in the environment, with infections among humans living in wildlife areas posing potential risks for transmitting pathogens, including ARB, to wild animals. Recent studies in Japan revealed a low prevalence of ARB in free‐living wild animals, depending on their proximity to human activities, feeding habits, behavioral patterns, and habitats (Asai et al., 2020; Tamamura‐Andoh et al., 2021). However, wild animals in urban parks (Ikushima et al., 2021) and around animal facilities serve as reservoirs of ARB (Yossapol et al., 2021). Therefore, wild animals in areas close to human activities may carry ARB transmitted from humans and domestic animals.

As AMR is a growing public health and pharmacological concern worldwide, it should be recognized that ARB in the excrement of wild animals may also pose a health risk to human beings. In this study, to clarify the possible health risk of ARB associated with wild animals living around humans, we analyzed fecal samples from unidentified animals and their feed habitats. We also estimated the prevalence of ARB in these unidentified animals.

## 2 MATERIALS AND METHODS

**Figure 1:**
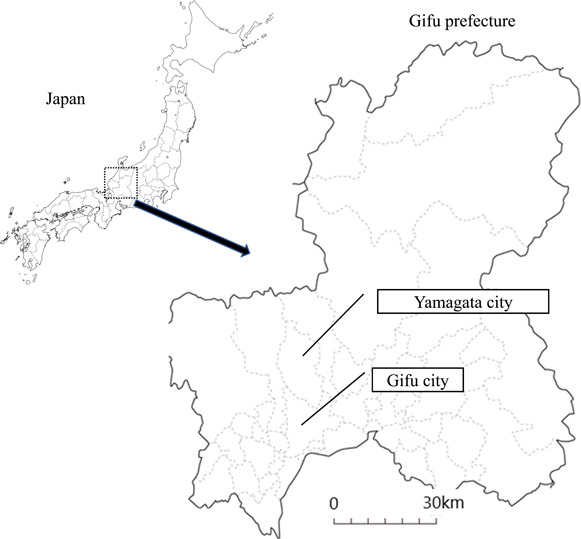
Locations of Gifu City and Yamagata City, Gifu Prefecture, Japan.

Eleven fecal samples from unidentified animals were collected around the parking space of a university (Gifu City, Japan), in the bushes around a pig farm (Gifu City), and in the garden close to a house (Yamagata City, Japan), as shown in Figure 1 and Table 1.

**Table 1:** Sample information, identified animal species, and resistance profiles of the *Escherichia coli* isolates

				Resistance profiles (no. of isolates)		
Sample ID	Date (y/m/d)	Location	Animal species	DHL	CTX‐DHL	NAL‐DHL
WL201209	2020/12/9	Parking space	*Vulpes vulpes*	Susceptible (3)		
WL210121	2021/1/21	Parking space	*Vulpes vulpes*	Susceptible (3)		
WL210405	2021/4/5	Parking space	*Vulpes vulpes*	Susceptible (2)	AMP‐CFZ‐CTX‐KAN‐TET (3)	AMP‐NAL‐CIP (3)
				AMP‐CEZ‐CTX‐KAN‐TET (1)		
WL210427	2021/4/27	Pig farm	*Martes melampus*	Susceptible (2)		
				KAN (1)		
WL210514‐1	2021/5/14	Pig farm	*Canis lupus familiaris*	Susceptible (3)		
WL210514‐2	2021/5/14	Pig farm	*Martes melampus*	Susceptible (3)		
WL210615‐1	2021/6/15	House garden	*Rhinolophus ferrumequinum nippon*	Susceptible (3)		
WL210615‐2	2021/6/15	House garden	*Felis catus*	AMP‐TET‐CHL‐SXT (3)		
WL210702	2021/7/2	Parking space	*Paguma larvata*	Susceptible (3)		
WL211207‐1	2021/12/7	Parking space	*Vulpes vulpes*			
WL211207‐2	2021/12/7	Parking space	*Vulpes vulpes*	Susceptible (3)	AMP‐CFZ‐CTX‐NAL‐CIP (1)	

*Note*: CTX‐DHL, DHL‐containing cefotaxime (1 mg/L); NAL‐DHL, DHL‐containing nalidixic acid (32 mg/L).

Abbreviations: AMP, ampicillin; CFZ, cefazolin; CIP, ciprofloxacin; DHL, deoxycholate hydrogen sulfide lactose agar; KAN, kanamycin; SXT, sulfamethoxazole/trimethoprim; TET, tetracycline.

For animal identification, fecal DNA was extracted from the samples using a QiaAmp Fast DNA Stool Mini Kit (ID: 51604; Qiagen) following the manufacturer's instructions, which was then subjected to a polymerase chain reaction (PCR) using a previously reported primer set (MiMammal‐U) (Ushio et al., 2017). The PCR products were sequenced at the Life Science Research Center of Gifu University, and the sequences were analyzed using a basic local alignment search tool (BLAST; National Center for Biotechnology Information). The animal species were identified based on genetic and ecological information.

*Escherichia coli* was isolated using deoxycholate hydrogen sulfide lactose (DHL) agar (Nissui Pharmaceutical) with or without antimicrobials (cefotaxime [CTX] 1 mg/L or nalidixic acid [NAL] 32 mg/L). Furthermore, bacterial species were identified using an API 20E bacterial identification system (BioMérieux). The minimum inhibitory concentration (MIC) was determined via broth microdilution, as previously described (Asai et al., 2020). Twelve antimicrobials, namely, ampicillin (AMP), cefazolin (CFZ), CTX, meropenem, gentamicin, kanamycin (KAN), tetracycline (TET), NAL, ciprofloxacin (CIP), colistin (CST), chloramphenicol (CHL), and trimethoprim/sulfamethoxazole (SXT) were tested. *Staphylococcus aureus* (ATCC29213) and *E. coli* (ATCC25922) were used for quality control during antimicrobial susceptibility testing (Clinical and Laboratory Standards Institute, 2022). The MICs of all the antimicrobials were analyzed based on the guidelines of the Clinical and Laboratory Standards Institute (2022).

Bacterial genomic DNA was extracted from bacterial cell suspensions inactivated with phenol/chloroform. Thereafter, the isolated DNA was subjected to bead‐beating in ZR BashingBead lysis tubes (Zymoresearch) for 10 min followed by vortexing and purification using a QIAquick PCR Purification Kit (Qiagen). After library preparation using the Nextera XT Library Prep Kit (Illumina), whole‐genome sequencing (WGS) was performed using the iSeq system (Illumina). After de novo assembly using CLC Genomics Workbench software (Qiagen), the assembled contigs were queried using MLST 2.0, SeroTypeFinder 2.0, FimTyper 1.0, and ResFinder 3.1 software provided by the Center for Genomic Epidemiology (http://www.genomicepidemiology.org). Next, the fimH30 isolates obtained were further classified according to their CIP susceptibility and H30Rx status via subclone‐specific PCR (Banerjee et al., 2013).

The extracted DNA from five fox fecal samples was amplified using MiMammal‐U for mammals (Ushio et al., 2017) and *rbcl* (Primer 3) for plants (Aziz et al., 2017). Thereafter, the PCR products were purified using an AMPure XP Kit (Beckman Coulter Inc.) and used to prepare sequencing libraries using Nextera XT Index Kit v2 (Illumina). Before sequencing, the library was purified via agarose gel electrophoresis using the Wizard® SV Gel and PCR Clean‐Up System (Promega Co.). The purified library samples were then sequenced using Illumina iSeq with 2 × 150‐bp paired‐end kits (Illumina), whereas the raw reads were analyzed using QIIME2. For this, the forward and reverse reads were merged, and low‐quality (less than 98%) tails were excluded. After the removal of both primer sequences from the assembled reads, quality filtering was performed to remove reads with an expected error rate of 1% or more and short reads of 100 bp or less. Amplicon sequence variants (ASVs) were generated, and denoising was performed using the “unoise” algorithm. Thereafter, ASV identification was performed using BLAST, based on a 98.5% homology criterion. When multiple species of plants appeared, they were further distinguished using YList (http://www.ylist.info/index.html) and published literature (Gifu‐ken Shokubutsushi Chosa‐kai, 2019).

## 3 RESULTS AND DISCUSSION

Of the 11 fecal samples, five (sample IDs: WL201209, WL210121, WL210405, WL211207‐1, and WL211207‐2) were identified as originating from the Japanese red fox, *Vulpes japonica* (100% similarity to *Vulpes vulpes*). Of the others, two (WL210427 and WL210514‐2) were identified as originating from Japanese martens (*Martes melampus*), one (WL210514‐1) from a stray or domestic dog (*Canis lupus familiaris*), one (WL210615‐1) from a greater horseshoe bat (*Rhinolophus ferrumequinum nippon*), one (WL210615‐2) from a stray or domestic cat (*Felis catus*), and one (WL210702) from a masked palm civet (*Paguma larvata*). Thus, the two samples identified as originating from a dog and cat may be samples from domestic animals. Except for the greater horseshoe bat, five of the identified species were small‐ to medium‐sized omnivorous mammals. In Japan, they are widely distributed in forests, bushes, and other areas inhabited by humans.

From the 10 excrement samples, 30 isolates of *E. coli* were obtained using DHL agar without antimicrobials. No isolates were obtained from sample WL211207‐1. One isolate, from a Japanese red fox (WL210405), exhibited resistance to AMP, CFZ, CTX, KAN, and TET; three isolates from a cat (WL210615‐2) showed resistance to AMP, TET, CHL, and SXT; and one isolate from a Japanese marten (WL210427) exhibited KAN resistance. The remaining 25 isolates were susceptible to all antimicrobials tested.

Using DHL containing 1 mg/L CTX, four *E. coli* isolates were obtained from two samples of Japanese fox (WL210405 and WL211207‐2) (Table 1). Of these, three isolates from WL210405 were resistant to AMP, CFZ, CTX, KAN, and TET, and one isolate from WL211207‐2 was resistant to AMP, CFZ, CTX, NAL, and CIP. Using DHL‐containing 32 mg/L NAL, three *E. coli* isolates were obtained from a fox sample (WL210405), and all these three isolates were resistant to AMP, TET, NAL, and CIP. Thus, bacteria resistant to medically important antimicrobials, that is, third‐generation cephalosporins and fluoroquinolones, were isolated from the two fox samples.

All four CTX‐resistant isolates obtained from sample WL210405 using DHL with and without CTX showed an identical pulsed‐field gel electrophoresis (PFGE) profile (see Appendix). The CTX‐resistant isolates were obtained using DHL agar without antimicrobials, indicating that the isolate was relatively dominant considering the presence of fecal *E. coli* in the foxes. Given that wild animals, including foxes, are rarely treated with antimicrobials, the isolates may be dominant in the intestines of foxes.

Next, given two CTX‐resistant isolates, one (wlxctx‐1) from sample WL210405 and the other (wlxctx‐4) from sample WL211207‐2 showed PFGE profiles identical to those as demonstrated by the abovementioned four isolates from WL210405, the two isolates were subjected to NGS analysis. WGS analysis revealed that the isolate from WL210405 (AMP‐, CFZ‐, CTX‐, KAN‐, and TET‐resistant) was O83:H42‐ST1485‐fimH58 CTX‐M‐55**‐**producing *E. coli* carrying the genes *aph(3″)‐Ib*, *aph(3′)‐Ia*, *aph(6)‐Id*, *mdf*(A), *sitABCD*, *sul*2, *tet*(A), and *tet*(B). Specifically, multiple genes, (*aph(3″)‐Ib*, *aph(3′)‐Ia*, and *aph(6)‐Id*) encoding aminoglycoside phosphotransferases present in this isolate were responsible for its KAN resistance, whereas both *tet*(A) and *tet*(B) were responsible for its TET resistance. The extended‐spectrum β‐lactamase (ESBL) producer was phenotypically and genotypically defined as multidrug‐resistant. Reportedly, CTX‐M‐15‐producing ST1485 *E. coli* has been observed in community dwellers in Japan (Nakamura et al., 2016). The other isolate from WL211207‐2 (with AMP, CFZ, CTX, NAL, and CIP resistance) was O25:H4‐ST131‐fimH30 CTX‐M‐14**‐**producing *E. coli* carrying the genes *mdf*(A) and *sitABCD*, with quinolone resistance‐determining regions mutations in *gyrA* (S83L, D87N), *parC* (S80I, E84V), and *parE* (I529L). Further analysis revealed that the O25:H4‐ST131 strain could be classified as H30R/non‐Rx. Reportedly, O25:H4‐ST131 *E. coli* is a pandemic clone that is disseminated among humans (Kawamura et al., 2018) and companion animals (Kawamura et al., 2017) in Japan and other countries (Banerjee & Johnson, 2014). Although CTX‐M‐27‐producing *E. coli* is dominant among ST131 H30R in humans and companion animals in Japan, CTX‐M‐14**‐**producing *E. coli* has also been reported in these populations (Kawamura et al., 2018, 2017). In this study, though both ESBL producers were first isolated from Japanese red foxes, they may have spilled over from humans and domestic animals, including companion animals. However, it does not appear to be a case of continuous carriage in fox(es), as identical ESBL producers were not isolated from two foxes sampled at almost the same location. Thus, identical ESBL producers were not isolated from foxes thereafter, implying that foxes may be transient carriers.

To elucidate the relationship between wild animals and human activity, the DNA metabarcoding method was applied to analyze the feeding habits of the five identified foxes. The presence of a domestic animal, domesticated plant, and forage crop genes in the feces of these wild animals implied possible direct or indirect connection with human activities (agricultural and animal industries). Regarding the fecal samples from the five animals, four contained 99% or more reads identified as fox genes, whereas one sample (WL210405) contained reads corresponding to fox (94.4%) and mole cricket genes (5.6%), as shown in Table 2. Although one sample (WL210121) showed the presence of a few dog‐related genes (0.5%), there was no evidence of association with livestock animals. The genes of fruits, such as oriental persimmons and fig trees, were detected in three samples (WL201209, WL211207‐1, and WL211207‐2) (Table 2). Notably, oriental persimmons are cultivated in Gifu, while some are wild. Unfortunately, these two types of persimmons could not be distinguished using the methods employed in this study. Additionally, the genes of domesticated plants, such as cucumber, sunflower, and rapeseed, were detected in WL210121. We also observed the genes of undomesticated plants in sample WL210405. Of the five fox samples studied, the hosts of two samples (WL210405 and WL211207‐2), from which the ESBL producer was isolated, fed on undomesticated plants and fruits, respectively. Even though there are no reports regarding food retention time in foxes, we reasoned that meals, including plant fiber, may be excreted within two days, as is the case in dogs (Burrows et al., 1982). The effects of seasonality and the issue of retention time duration should be fully considered in the subsequent studies. As different types of ESBL producers were found at the same location, it is also necessary to consider the excretion period of the resistant bacteria. Since this study did not observe the intestinal contents associated with domestic animals and plants in the two foxes carrying the ESBL producers, it was impossible to infer the origin of the ARB they carried.

**Table 2:** Animal and plant genes were detected in fecal samples from Japanese red foxes

Sample ID	Animals: Proportion	Plants: Proportion
WL201209	*Vulpes vulpes* (red fox): 99.9%	*Diospyros kaki* (*Oriental persimmon*): 85.9% *Ficus carica* (fig tree): 10.6% *Broussonetia papyrifera* (*Paper mulberry*): 3.4%
WL210121	*Vulpes vulpes* (red fox): 99.5% *Canis lupus familiaris* (dog): 0.5%	*Cucumis sativus* (cucumber): 51.9% *Helianthus annuus* (sunflower): 14.0% *Festuca arundinacea* (tall fescue): 11.6% *Oxalis stricta* (yellow wood sorrel): 10.1% *Brassica napus* (rapeseed): 5.1%
WL210405	*Vulpes vulpes* (red fox): 94.4% *Gryllotalpa orientalis* (mole cricket): 5.6%	*Vicia tetrasperma* (hairy tare): 70.3% *Cardamine hirsuta* (*Hairy bittercress*): 14.3% *Cerastium glomeratum* (sticky chickweed): 6.7% *Stellaria graminea* (common starwort): 4.0%
WL211207‐1	*Vulpes vulpes* (red fox): 99.9%	*Aphananthe aspera* (muku tree): 90.2% *Diospyros kaki* (*Oriental persimmon*): 3.5%
WL211207‐2	*Vulpes vulpes* (red fox): 99.2%	*Diospyros kaki* (*Oriental persimmon*): 75.0% *Aphananthe aspera* (Muku tree): 20.7%

## 4 CONCLUSION

Taken together, this study identified ESBL‐producing *E. coli* in Japanese red foxes for the first time. This finding suggests that appropriate and careful handling of feces dropped around areas of human activities is essential. Notwithstanding, this study had some limitations. For example, it was conducted using a small number of fecal samples from wild animals. Therefore, continuous estimation of AMR pollution among wild animals is required as ESBL producers may spill over from human activities to wild foxes.

## AUTHOR CONTRIBUTIONS

**Tetsuo Asai**: Conceptualization (lead); writing – original draft, review, and editing (lead); and formal analysis (equal). **Michiyo Sugiyama**: Formal analysis (lead). **Tsutomu Omatsu**: Formal analysis (supporting) and review and editing (supporting). **Masato Yoshikawa**: Formal analysis (supporting) and review (supporting). **Toshifumi Minamoto**: Formal analysis (supporting) and review and editing (supporting).

## CONFLICT OF INTEREST

None declared.

## ETHICS STATEMENT

None required.

## Data Availability

The datasets of *E. coli* from WL210405 and WL211207‐2 generated and analyzed during the current study are available in the NCBI database, accession numbers: DRA014430 (https://www.ncbi.nlm.nih.gov/sra/DRA014430) and DRA014431 (https://www.ncbi.nlm.nih.gov/sra/DRA014431).

## 1

Xbal‐digested pulsed‐field gel electrophoresis (PFGE) profiles of four cefotaxime (CTX)‐resistant *Escherichia coli* isolates from a Japanese wild fox (WL210405) fecal sample. PFGE was performed using the XbaI restriction enzyme, following the PulseNet protocol (Ribot et al., 2006). Lane M, *Salmonella enterica* serovar Braenderup H9812 digested with XbaI; Lanes 1–3, three isolates (wlxctx‐1, wlxctx‐2, and wlxctx‐3) obtained using CTX‐containing deoxycholate hydrogen sulfide lactose agar; and Lane 4, one isolate (wlx‐16) obtained using nonantimicrobial containing agar.
